# An optimized chronology for a stalagmite using seasonal trace element cycles from Shihua Cave, Beijing, North China

**DOI:** 10.1038/s41598-018-22839-z

**Published:** 2018-03-14

**Authors:** Fengmei Ban, Andy Baker, Christopher E. Marjo, Wuhui Duan, Xianglei Li, Jinxian Han, Katie Coleborn, Rabeya Akter, Ming Tan, Gurinder Nagra

**Affiliations:** 1 Faculty of Environmental Economics, Shanxi University of Finance & Economics, Taiyuan, 030006 China; 2 Connected Waters Initiative Research Centre, UNSW Sydney, Sydney, NSW 2052 Australia; 3 Mark Wainwright Analytical Centre, UNSW Sydney, Sydney, NSW 2052 Australia; 4 Key Laboratory of Cenozoic Geology and Environment, Institute of Geology and Geophysics, Chinese Academy of Sciences, 100029 Beijing, China; 5 Institute of Global Environmental Change, Xi’an Jiaotong University, Xi’an, 710049 China

## Abstract

Stalagmites play an important role in paleoclimatic reconstructions from seasonal to orbital time scales as ^230^Th-dating can provide an accurate absolute age. Additionally, seasonal trace element and optical layers can provide a precise age. We analyzed the seasonal variability of multiple trace elements on a stalagmite (XMG) in Shihua Cave, Beijing and compared them with results from laminae counting. The results show that (1) the polished section of the topmost part of XMG has obvious bi-optical layers under a conventional transmission microscope, however, laminae are not observed using this method in the rest of the sample, and (2) The variations of P/Ca, Sr/Ca, Ba/Ca, U/Ca and Mg/Ca show seasonal cycles throughout the sample. The PC1 in the Principal Component Analysis (PCA) of five trace elements represents the annual cycle. This stalagmite was deposited over 150 ± 1 years through PC1 peak counting. This result corresponds well with the annual layers and U-Th dating. Trace element cyclicity of PC1 can increase the accuracy of stalagmite dating, especially in the absence of obvious laminae and are a powerful method to identify seasonal changes in a strongly contrasting wet-dry monsoon climate region.

## Introduction

Stalagmites are increasingly important for paleoclimatic reconstruction because of their wide distribution, multiple proxies, high resolution and precise dating. Especially, the timescale over which it is possible to build paleoclimate reconstructions can be from seasonal to orbital timescales^1,2^. Trace element variations of stalagmites have been used to explore sub-annual climatic and cave environmental signals and for chronology building^1,3–11^. With the development of *in-situ* measurements, high resolution sampling methods such as secondary ion mass spectrometry (SIMS)^1,3,8–10,12^, laser-ablation inductively coupled plasma mass spectrometry (LA-ICP-MS)^4–6,11,13,14^ and micro-X-ray fluorescence imaging^15,16^ have been used to measure trace elements to get annual and even seasonal signals, which can be used to explore sub-annual climatic, cave environmental signals and chronology building.

Because of a seasonally changing climate, such as wet and dry seasons, and associated seasonal changes in hydrological or ventilation conditions within the cave^17–19^, some stalagmites show obvious annual laminae under visible-light or UV-fluorescence, which can be used to obtain an accurate age by counting laminae and comparison to radiometric methods^20–25^. Some stalagmites fail to exhibit visible or UV-fluorescence laminae, or they lack laminae in some sections, but the same stalagmites may show cycles in their trace element composition^1^. The impact of seasonality on speleothems would be expected to be strong enough to generate annual chemical variations, whether or not conventional growth laminae are visible^26,27^. In 2009, Smith *et al*.^10^ built an objective and automated technique for establishing trace element chronologies analysing individual elements in stalagmites from two Alpine caves. Then Nagra *et al*.^11^ exploited seasonal variations in trace elements to construct chronologies in a Mediterranean climate employing principal components analysis (PCA). These studies imply that cyclicity of trace elements can be used to obtain an accurate age for non-laminated speleothem samples.

In Shihua Cave, stalagmites typically have obvious laminae observed under visible-light and UV-fluorescence^28^, and the annual laminae thickness, the isotope of oxygen and carbon and trace elements have been used to reconstruct past climate and paleoenvironmental signals^25,29^, including a 2650-year annual resolution record of warm season temperature^21^. Observations of drip water and calcite deposition in this cave shows that opaque layers (the fluorescent layer) was formed by the pulses of soil organic matter in the rainy season, while the thick calcite layer was formed in the dry season^19,30^.

Stalagmite-XMG was growing when it was collected in 2015. U-Th dating has demonstrated that it grew for around the last 145 years^25^. Under the confocal fluorescent microscope, it shows clear fluorescent cycles throughout the stalagmite transect, and 143 ± 7 cycles were counted^25^. Its oxygen isotopes have been interpreted as recording East Asian Summer Monsoon (EASM) variability over the last 145 years^25^. In this study, we aim to establish the optimal chronology for stalagmite XMG by comparison between the PCA of multiple trace element ratios (P/Ca, Sr/Ca, Ba/Ca, U/Ca and Mg/Ca) and the stalagmite fluorescent laminae. These comparisons allow us to evaluate the accuracy of different dating techniques and test whether the trace elements can supply the similarly accurate chronology. It is important to build stalagmite chronology by using annual trace element variability of multiple trace elements in a monsoonal climate. Additionally, we investigate whether individual climatically-sensitive elements in the stalagmite, such as P and Sr, can relate to dry and wet signals and have the potential for paleoclimate reconstruction.

## Site Description

Shihua Cave (115°56′E, 39°47′N, 251 m above sea level at the entrance) is located in Baihua Hill, Fangshan county, about 50 km away from southwest Beijing. Shihua Cave is situated in the Middle Ordovician carbonate rocks of the Maojiagou Formation, which mainly consists of limestone, with some interlayered dolomite. This limestone strikes in an east-west direction and is inclined towards the south at about 30°. The thickness of the bedrock overlying the cave varies between 30 and 130 m. Coal layers are unevenly distributed in the overlying bedrock. Today, the natural vegetation above the cave is dominated by shrubs, while persimmon and walnut trees are grown in the valley. The overlying soil is brown soil and its thickness varies from 0.6 to 1.0 m in the valley and 0 to 0.5 m on the hill slopes. The soil above the cave consists of a 5–15 cm humic layer that has a high content of total organic carbon (40–70 g·kg^−1^).

Situated within the East Asian monsoon zone, the Shihua Cave region typically has cold/dry winters and warm/wet summers. The East Asian monsoon is the major source of precipitation. In Beijing more than 70% of the total annual precipitation falls during the summer monsoon months (June to August), which are also the warmest months. The dry period lasts for at least 8 months from October to May of the next year. From 1971–2000 CE, the mean annual temperature was 12.3 °C while mean annual precipitation was 572 mm.

**Figure 1:**
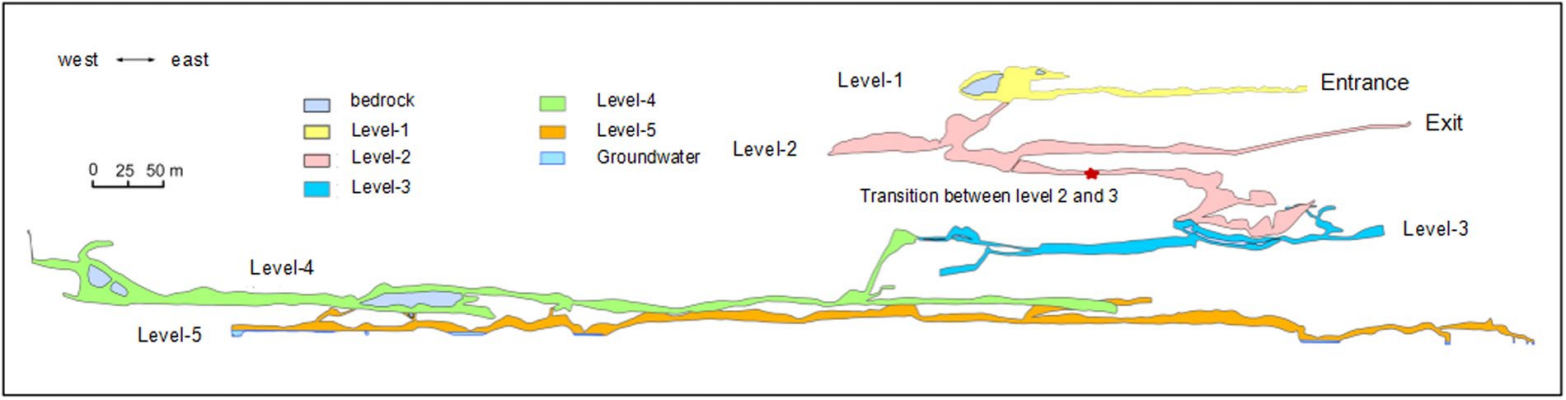
Profile map of Shihua Cave (after Liu Hong *et al*., 2013) and the location of stalagmite XMG (red star). Figure used by permission from Cai Binggui.

Shihua Cave has a multi-level and multi-branched structure. Based on the newest survey, the cave has five levels^31^ (Fig. 1). The passages open to visitors (first to third levels) are about 2500 m long.

The XMG stalagmite, with a diameter of 40 mm and height of 36 mm, was located in the transition level between the level 2 and 3 (See Fig. 1). It was confirmed that it was actively growing when we collected it in October 2015 through the monitoring of calcite deposition on a glass plate for half a year. A total of 143 annual fluorescent layers have been previously counted with a seven year error at the bottom, consistent within the margin of error of the ^230^Th analyses^25^.

## Results

### XMG stalagmite laminae

**Figure 2:**
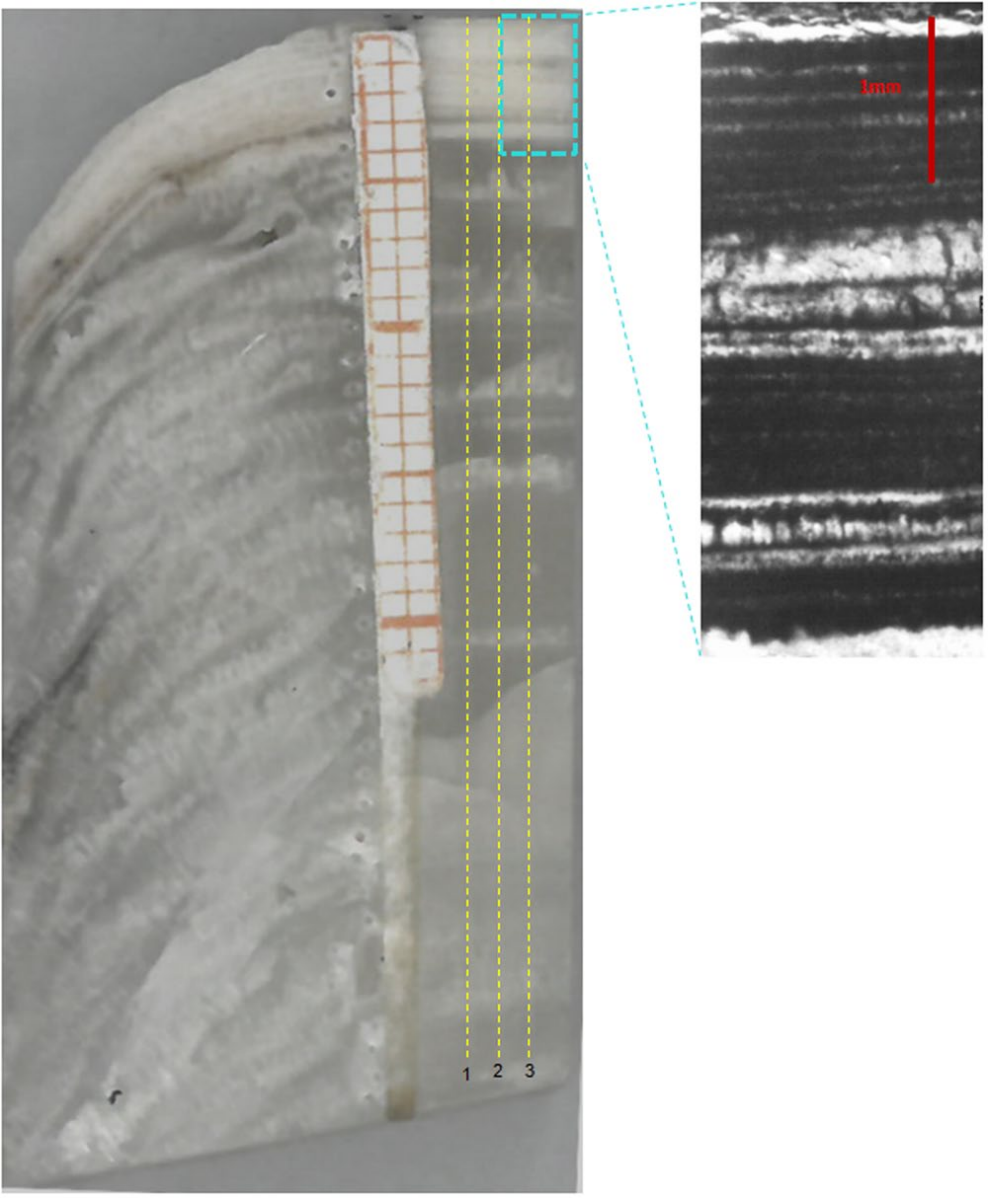
Stalagmite XMG images collected during this study showing the sampling transects used for LA-ICP-MS analysis and (inset expanded on the right) micrograph of stalagmite XMG showing the contrast in topmost layers revealed by white-light illumination.

The XMG stalagmite comprises calcite that has obvious layers under conventional visible-light and mercury-source ultra-violet transmission microscopy, but only in the topmost part. From the top to 3.8–4 mm, the variability in depth is due to uneven growth layer thickness(Fig. 2).These layers are similar to other stalagmites from the cave^21,28,29^, where the laminae have been described as bi-optical (the interfaces seem dark under transmitted light and luminescent under reflected and ultra-violet light)^21,28^. There are no layers apparent using visible-light or mercury-source ultra-violet transmission microscopy in the rest of XMG using the same method. There is also a significant difference in the character of the laminae in the topmost part of XMG and previously published stalagmites in Shihua Cave. Stalagmite XMG has a thick opaque layer and a thin transparent layer under conventional transmission microscopy, while previously published stalagmites records comprised thin dark layers and thick transparent layers^19,21,29^. In most stalagmites from the cave, the thin interfaces of the laminae consist of opaque calcite with the presence of organic matter.

In stalagmite XMG, using mercury-source ultra-violet transmission microscopy, a total of 23 visible and fluorescent layers were counted in the top 3.8–4.0 mm, with an accumulated error of 1 year. Assuming that these are annual, this indicates that the deposited calcite fabric on XMG has changed since the early 1990s. This could be related to the cave environment, as an artificial exit was created in 1987 when the cave was opened to tourists (see Fig. 1). Although no data exists to verify this hypothesis, we suggest this exit has likely changed ventilation and cave air CO_2_ concentrations. The XMG stalagmite was located on the transition between levels 2 and 3, where the passage is narrow. Ventilation patterns are likely to have changed here due to seasonal changes in ventilation between the two the entrances at different elevations induced by the chimney effect^32^.

Under the confocal fluorescence microscope, stimulated with a 40 mW laser (488 nm wavelength), and using a filter which isolated the emitted wavelengths at 515/30 nm—the green portion of the visible spectrum, it has been previously shown that clear fluorescent laminae are present throughout the whole stalagmite^25^. This instrument has better resolution, image sectioning and signal-to-noise ratio than the traditional fluorescent microscope^33^. It is this confocal fluorescence microscope data that is used in comparison with trace element data.

### Trace element chronology

**Figure 3:**
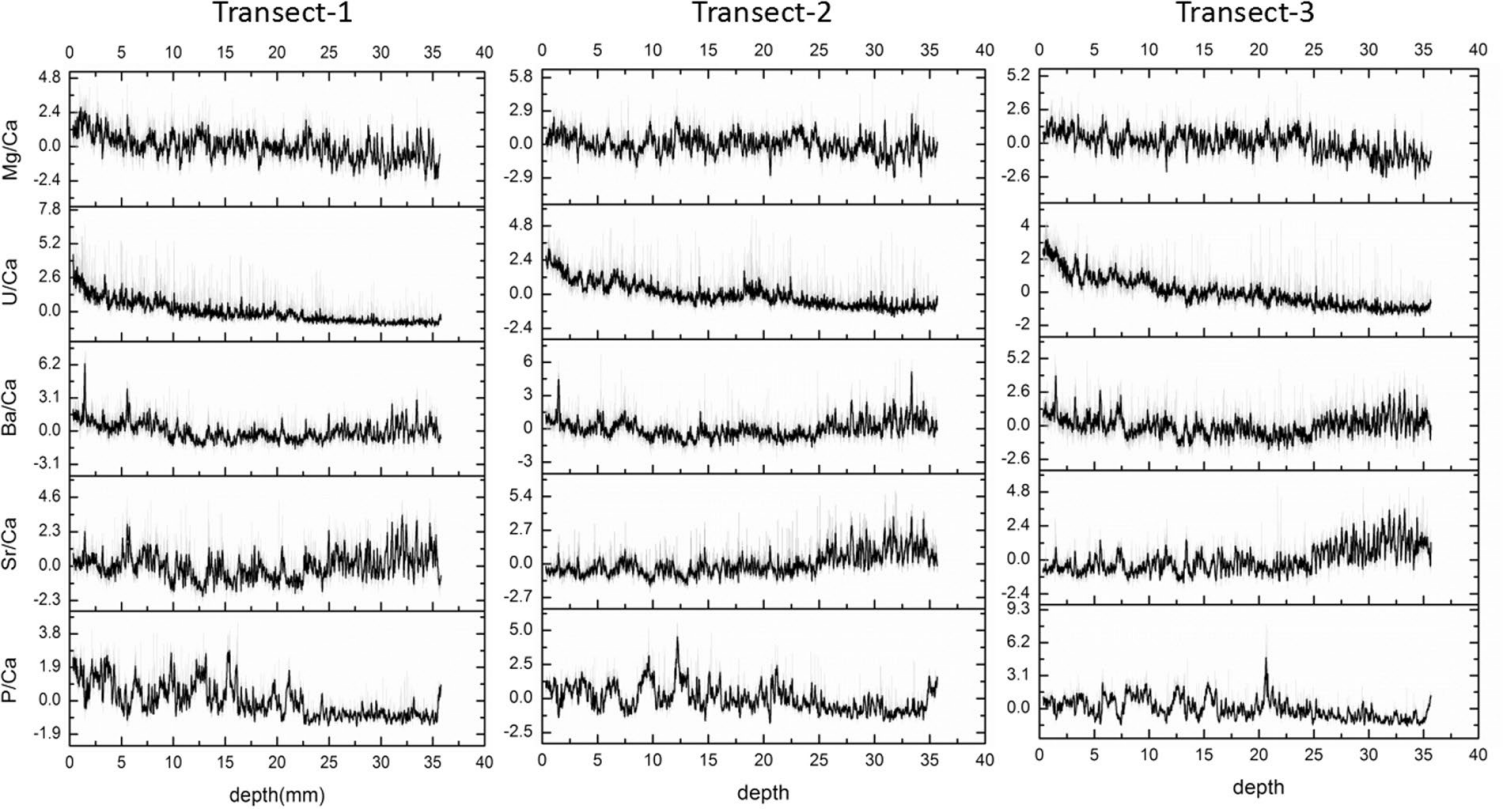
The variation of P/Ca, Sr/Ca, Ba/Ca, U/Ca and Mg/Ca in the three transects. The similar annual cycles of smoothing lines are showed (17 point window Savitzky-Golay method).

**Figure 4:**
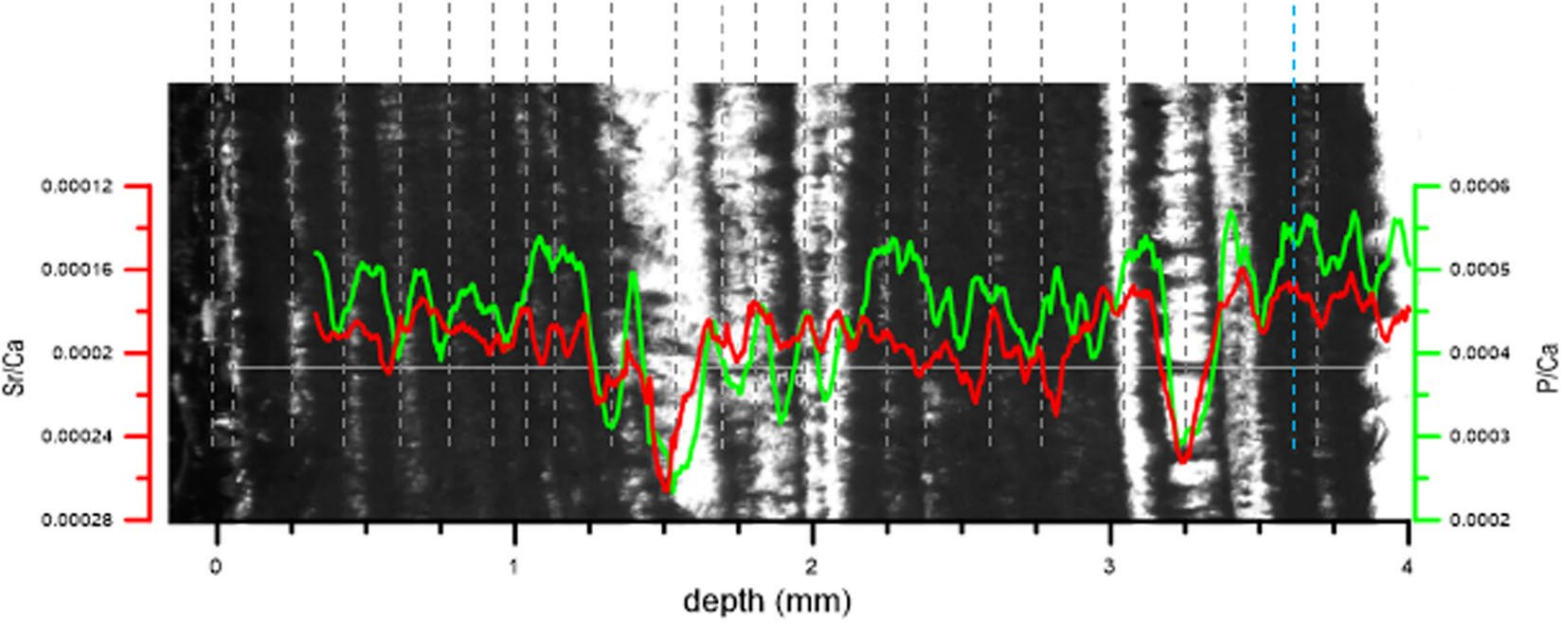
Comparison between the peaks of Sr/Ca and P/Ca and layers in transect 3. The vertical dashed lines represent the transparent laminae formed in dry seasons (the blue dashed line indicates uncertain lamina). Note the y-axis of Sr/Ca is inverted, so that the peaks of Sr/Ca in the graph corresponds to the lower value of Sr/Ca. Stalagmite XMG images were collected during this study.

Figure 3 shows the trace element time-series for P/Ca, Sr/Ca, Ba/Ca U/Ca, and Mg/Ca, using a 17-point Savitzky-Golay smoothing (Supplementary Fig. S1 includes raw data). We observe similar annual cycles in all the elements (Fig. 3). Table 1 shows that Sr/Ca is inversely correlated to P/Ca (Table 1). When Sr/Ca and P/Ca are compared with the visible-light lamina image, we can see that the peaks of Sr/Ca correspond with the white layers and the peaks of P/Ca match closely with the opaque layers (Fig. 4). In Shihua Cave, the white layer (low-organic calcite) deposits on the stalagmites in late winter and spring when the climate is dry, then the opaque layer (organic-rich calcite) is formed in the high rainfall monsoon period^19,30^. This can confirm P is a soil-derived element, which increases in the high rainfall monsoon period.

**Table 1:** The Pearson correlation coefficient of five trace elements in three transects.

	P/Ca	Sr/Ca	Ba/Ca	Mg/Ca	U/Ca
**Transect-1**					
P/Ca	1				
Sr/Ca	−0.36*	1			
Ba/Ca	−0.01	0.70*	1		
Mg/Ca	0.45*	−0.07*	0.21*	1	
U/Ca	0.55*	0.02	0.35*	0.43*	1
**Transect-2**					
P/Ca	1				
Sr/Ca	−0.55*	1			
Ba/Ca	−0.32*	0.64*	1		
Mg/Ca	0.34*	−0.33*	0.11*	1	
U/Ca	0.34*	−0.24*	−0.07*	0.23*	1
**Transect-3**					
P/Ca	1				
Sr/Ca	−0.59*	1			
Ba/Ca	−0.28*	0.58*	1		
Mg/Ca	0.42*	−0.39*	0.17*	1	
U/Ca	0.51*	−0.48*	−0.13*	0.45*	1

*Correlation is significant using 2-tailed test at the 0.05 level.

**Figure 5:**
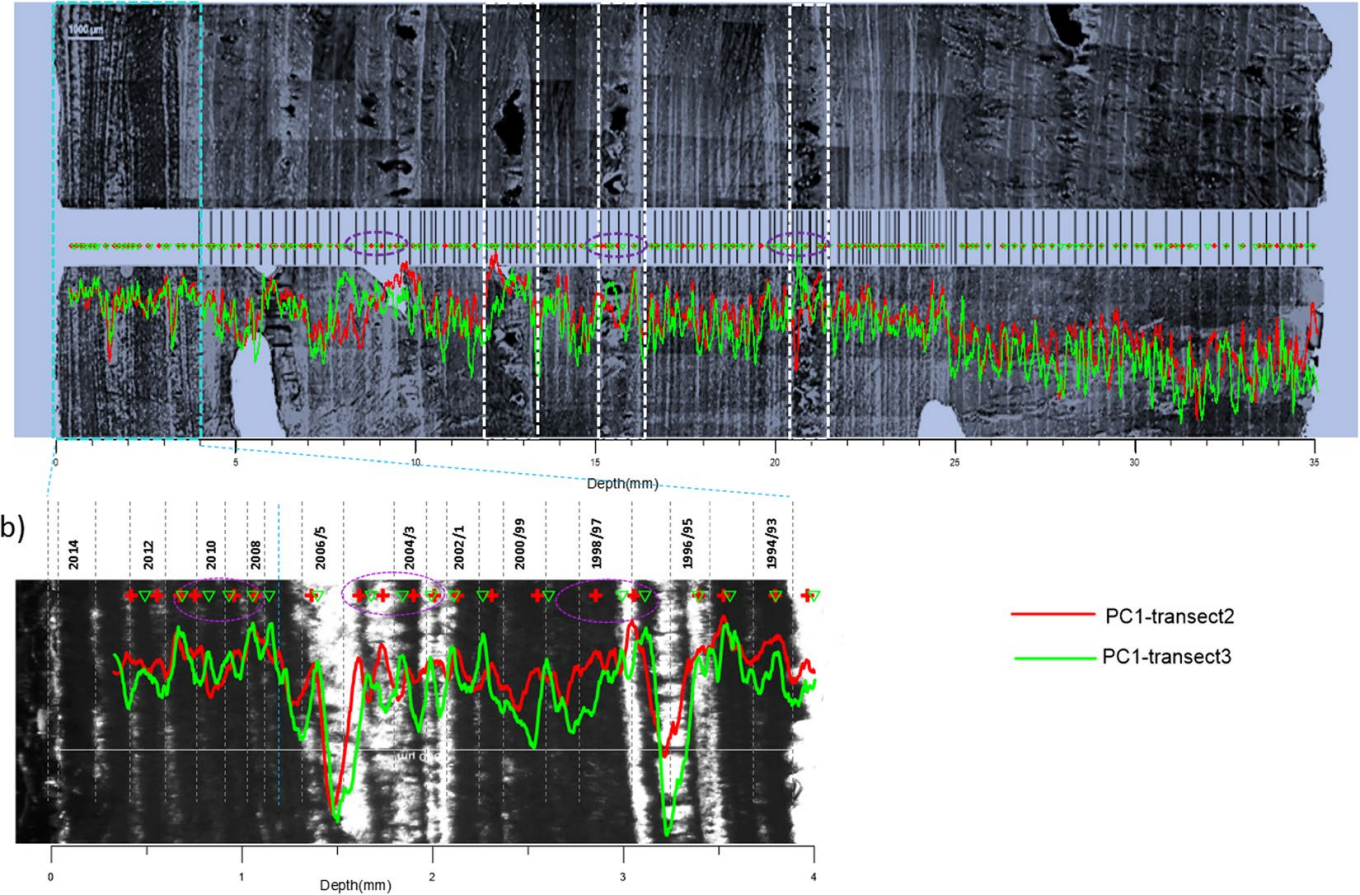
The comparison between the laminae images (revised from Li *et al*. 2017 by recoloring and adjusting the brightness and contrast of image using the Microsoft Powerpoint 2010) and the two PC1. (Purple ellipses means the location of peaks are different between the two transects; white square area represent the locations without clear fluorescent layers). The numbers of b) are calendar age in which the layers were formed.

From Table 2, we can see there is close agreement in the number of peaks between the transect 2 and 3 (155 and 153 peaks respectively). In these two transects, PC1 has main contributions from Sr/Ca and P/Ca (Table 3). In contrast, transect 1 has 169 peaks for PC1 representing the changes in U/Ca and P/Ca. Transect 1 also has a higher number of peaks of Sr/Ca, and appears to include more sub-annual peaks compared to the other transects. By analyzing three tracks, we can judge that the PC1 in the transect 2 and 3 are comparable and can represent the annual cycle and be used to build the chronology. This result demonstrates the advantage of measuring at least three transects to enable rejection of transects that are less representative of annual trace element periodicity. After using the auto-counting method, further screening of the peaks was done by comparing the transects 2 and 3. Eight PC1 peaks in transect 2 and seven in transect 3 were removed manually (see Methods, Supplementary Fig. S2). After these peaks were removed, 147 peaks and 146 peaks are preserved respectively in transect 2 and 3. Because the first peak of trace elements measurement is on the fourth laminae from top (Fig. 5b), the XMG stalagmite grew for 150 years with a 1 year error based in its trace elements chronology

**Table 2:** The results of auto-counting peak based on three transects.

	PC1	PC2	P/Ca	Sr/Ca
Transect-1	169	165	166	166
Transect-2	155	194	165	153
Transect-3	153	186	161	153

**Table 3:** The loadings of each element onto the first two PCs from the PCA. PCs are listed which explain around 70% of the variance in each transect.

		P/Ca	Sr/Ca	Ba/Ca	U/Ca	Mg/Ca	variance%
transect1	PC1	**0.772**	−0.030	0.400	**0.847**	**0.759**	41.021
	PC2	−0.422	**0.940**	**0.846**	0.070	−0.058	35.718
transect2	PC1	**0.798**	**−0.875**	−0.631	0.475	0.499	45.513
	PC2	0.133	0.238	**0.699**	**0.675**	0.444	24.302
transect3	PC1	**0.821**	**−0.865**	−0.468	0.601	**0.744**	51.126
	PC2	0.045	0.275	**0.833**	0.652	0.267	25.346

To verify the chronology of the trace elements in the stalagmite, the peaks of PC1 in the transect 2 and 3 are compared with the fluorescent microscope image of the laminae from Li *et al*.^25^ (Fig. 5a). In the topmost part of the figure, we compare the transparent image with PC1 (Fig. 5b). Using microscopy, a total of 23 annual layers were counted with an error of 1 year. Due to the start position of the laser ablation transects, the first trace element peaks occur on the fourth lamina. The elemental chronology shows 19 and 18 peaks respectively in transects 2 and 3, which matches the microscopy layer counting very well. The absolute position of peaks in the two transects has a small difference because of uneven deposition rates across the surface (Fig. 5 purple ellipse). Through this comparison, the annual nature of the trace elements can be verified from their good agreement. Importantly, the measurement of trace elements is consistent even in places without obvious laminae (Fig. 5 white square). Therefore, the trace elements not only confirm the annual nature of the laminae but also provide a good alternative for stalagmites without obvious laminae.

**Figure 6:**
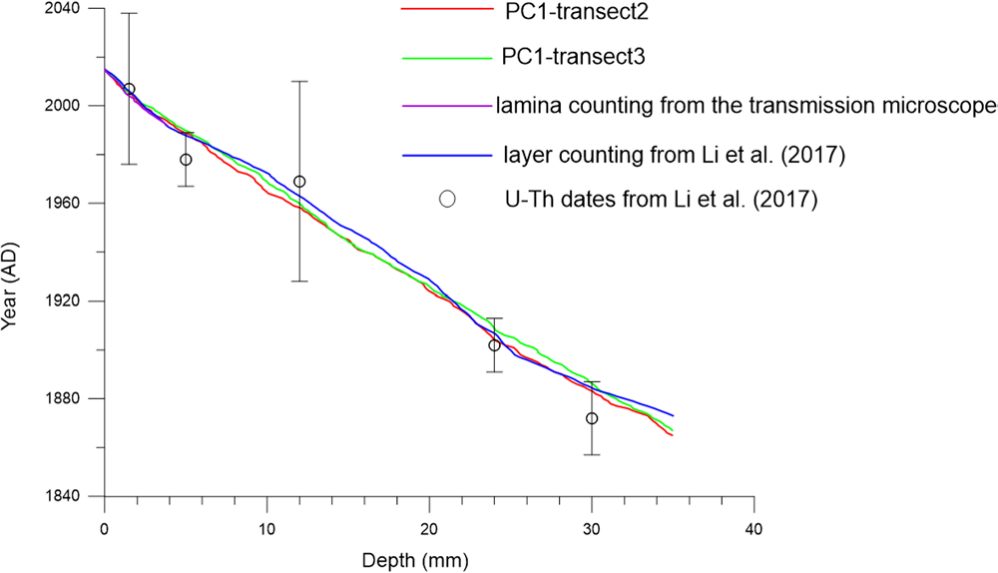
Age model of stalagmite XMG.

Despite small variations in the absolute peak location determined by trace element peak counting and microscopy, the distance between peaks can be used to determine an annual growth rate. In stalagmite XMG, the growth rate is faster below 25 mm from the top (0.26 mm/year) than above it (0.22 mm/year). The cumulative annual growth rate data can be used to derive age-depth relationships. Figure 6 shows that the age models of PC1 for the two transects are within the error of dating by U-Th and visible lamina counting. The PC1 variations in two transects are not synchronous, a result of uneven deposition perpendicular to the growth axis.

## Discussion

The regular alternation of dry and wet season climate, and the seasonal flush of rain can control the hydrological and hydrochemical properties of the drip water that feeds stalagmites, and can cause the annual cycles of trace elements. Seasonal variations have previously been observed in the trace elements in stalagmites from Mediterranean and Alpine climate areas, which also have a strong seasonality^4,11,12^.

In monsoonal climates, trace elements show seasonal cycles because of the dominant seasonal control of hydrology, which affects infiltration and prior calcite precipitation (PCP). During dry periods, the residence of recharge water in a karst aquifer can be long enough to result in high trace element-to-calcium ratios such as Sr and Ba because of PCP^34^. In Shihua Cave, less than 30% annual rainfall is in the dry period. During this period, most drip water comes from water which was recharged to the unsaturated zone in the preceding rain season, and due to PCP has higher Sr [Ba]/Ca. This contrasts with tropical climates, such as at Gunung Mulu and Gunung Buda National Parks in northern Borneo, where stalagmite Mg [Sr]/Ca ratios are poor indicators of hydroclimate conditions, due to a wetter climate and less strong seasonality^35^.

Phosphorous, as a nutrient element, is associated with soil and vegetation decay on the surface. It has been shown to have an association with colloid-associated metals e.g. Zn, Pb in cave drip waters^12,26,36^. At Shihua Cave, in the wet monsoon period, an increase in P/Ca is observed. The increase in P/Ca observed in stalagmite XMG, is indicative of a flush of soil-derived water in the summer monsoon. In Table 2 the P/Ca peaks are slightly greater than the number of PC1 peaks, suggesting that in some instances a sub-annual flush of soil derived material is possible. In Shihua Cave, field monitoring showed that intra-annual variability in drip discharge was significantly higher than inter-annual variability. The flushing peak of soil organic matter occurs above a threshold rainfall intensity, and can have several annual pulses corresponding with intense rain events^30^. The fact that the amount of P/Ca peaks is more than the annual cycles can be explained by multiple flushes in a year. Therefore, P/Ca can be as the signal of the long-duration and/or high-magnitude precipitation events.

Sr/Ca is strongly negatively correlated with the P/Ca in the XMG stalagmite and positively with Ba/Ca in Shihua Cave (Table 1) and this has been observed in previous studies^10,12^. The strong similarity between the variation of Sr/Ca and Ba/Ca, as also observed the Heshang Cave^6^, suggests a common control mechanism, namely PCP. Sr and Ba come from the overlying limestone, and the unsaturated zone water residence time may also be important for these elements on inter-annual scales.

Mg may have multiple sources in Shihua Cave. At the site the soil is derived both from bedrock weathering and wind-blown material. The latter includes clay which can be a significant source of Mg, as has been observed in regions of wind-blown dust deposition^37^. Soil mineral derived elements therefore have a complex origin that can change over time, as it can be influenced by the degree of clay weathering (temperature and humidity), its dissolution rate, as well as unsaturated zone water residence time (PCP). Mg/Ca, with a positive relation with U/Ca, P/Ca, and an anti-correlation with Sr/Ca, shows it has a closer relation with soil than bedrock.

In stalagmite XMG, U is complicated to interpret as it is organic-transported, but has to compete with other organic and colloidal bound elements. It may also come from both a soil and bedrock source. It is a complicated element for paleoclimate interpretation at our site to explain. The further monitoring field work should be carried to confirm its transferring mechanism.

The strong annual variation of key trace elements, can provide chronological markers for high-resolution studies of the climate proxies. Regular and continuous annual variations in trace element composition for PC1 and Sr/Ca in stalagmite XMG are apparent. Sufficient mixing of infiltration waters in the karst before reaching the cave must occur, which smooth much of sub-annual hydrological variability but retains sufficient seasonal variability. Stalagmite XMG, situated at a depth of ~60 m below the surface, has a hydrological connectivity sufficient to resolve this seasonality in trace elements. Seasonality in trace elements in drip waters and speleothems have been observed at shallower sites e.g. ~4 m below surface^11^, ~20 m below surface^12^ and ~30 m below surface^4^. This contrasts to other deeper sites, where no annual variation of trace elements has been observed e.g. over 1000 m depth at Corchia Cave of central Italy^38^.

Overall, the variations of five trace element-to-calcium ratios (P/Ca, Sr/Ca, Ba/Ca, U/Ca and Mg/Ca) show the seasonal cycle from the XMG stalagmite in Shihua Cave, located in East Asian monsoon zone. The PCA of the five trace elements showed that the cycles of PC1 represents the annual cycle. A PCA approach is beneficial for identifying the best elements sensitive to PCP and the annual hydrological cycle, and these can then be used as a record of annual laminae in the China monsoon area. We recommend P and Sr as the least ambiguous for use in paleoclimate in the Chinese monsoon regions. Sr cycles are closest to the annual cycles, and P provides a record of soil-connectivity, which may also include sub-annual climate information. Importantly the annual laminae of trace elements cover the shortage of unclear layer-counting. Therefore, the trace elements not only confirm the annual nature of the laminae but also provide a good alternative for stalagmites without obvious laminae.

## Methods

### Trace element measurements

The XMG stalagmite thick-section for trace elements analysis was cut with a wire line saw along its growth axis. The LA-ICP-MS analysis was completed at the Mark Wainwright Analytical Centre of the University of New South Wales, using NWR213 Laser Ablation sample accessory (ESI, USA) and a NexION 300D ICP-MS (PerkinElmer, USA). The laser wavelength was 213 nm operating at a frequency of 10 Hz at 70% power, equivalent to a fluence of 11.83 J/cm^2^, focused down to 80 μm on the sample, with a scan speed of 10 μm/sec along the transect. Two standards, NIST612 (Sr: 78.4, Ba: 41.0, P: 39.1, Mg: 85.09, and U: 37.38ppm) and NIST610 (Sr: 515.5, Ba: 453, Mg: 457 and U: 461.5 ppm) were used to calibrate the data before and after each scan transect.

To account for uneven growth, three parallel transects separated by 1 mm (Fig. 2) along the central growth axis of the sample were chosen to reduce within stalagmite variability in our measurements.

### Trace elements cycles counting

All trace data were ratioed to the calcium signal and standardised. The Pauta criterion was used to remove the outliers that coincided with small holes on the sample surface. The missing data was then interpolated using the average of neighbouring data.

Principal component analysis (PCA) can be used to identify components that explain the variability of all trace elements along the transect. Variation of the first component score along the transect should be related to the annual periodicity. The PCA/peak counting method for building chronologies will be most effective if only those elements that have the clearest cycles are included^11^. We chose the five trace elements (Sr/Ca, P/Ca, Mg/Ca, U/Ca and Ba/Ca) among the stalagmites which showed the annual variability then employed PCA using free software named Past3.x (download from https://folk.uio.no/ohammer/past/) in each transect to calculate the first principal component (PC1) that was associated with annual changes in trace element concentration.

The scores plots for PC1 along the transect was smoothed using the Savitzky-Golay method using a 17-point window then an automated peak counting program, ‘Peak Analyser’ in Originlab12 was used to locate peaks. A 25 point filter window was selected based the half of growth speed (~0.23 mm/year) to avoid missing annual peaks.

To the automated peak counting process was refined manually by identifying cross-calibration between two transects by applying the criteria, peaks that only emerged in one transect with a height less 30% of the neighbouring relative peaks were removed.

## Electronic supplementary material

Supplementary information
