# Supplementary material for: An optimized chronology for a stalagmite using seasonal trace element cycles from Shihua Cave, Beijing, North China
Source: Sci Rep. 2018 Mar 14;8:4551. doi: 10.1038/s41598-018-22839-z (PMC5852128; doi:10.1038/s41598-018-22839-z)
Supplement: Supplementary file 1 — Supplementary information [file 41598_2018_22839_MOESM1_ESM.pdf]

An optimized chronology for a stalagmite using seasonal trace element cycles from Shihua Cave, Beijing, North China

Fengmei Ban[1, 2*], Andy Baker[2], Christopher E. Marjo[2, 3], Wuhui Duan[4], Xianglei Li[5], Jinxian Han[1], Katie Coleborn[2], Rabeya Akter[3], Ming Tan[4], Gurinder Nagra[2]

1. Faculty of Environmental Economics, Shanxi University of Finance & Economics, Taiyuan, 030006, China

2. Connected Waters Initiative Research Centre, UNSW Sydney, Sydney, NSW, 2052, Australia

3. Mark Wainwright Analytical Centre, UNSW Sydney, Sydney, NSW 2052, Australia

4. Key Laboratory of Cenozoic Geology and Environment,Institute of Geology and Geophysics, Chinese Academy of Sciences, 100029, China

5. Institute of Global Environmental Change, Xi'an Jiaotong University, Xi'an, 710049, China

* corresponding author: banfm@163.com

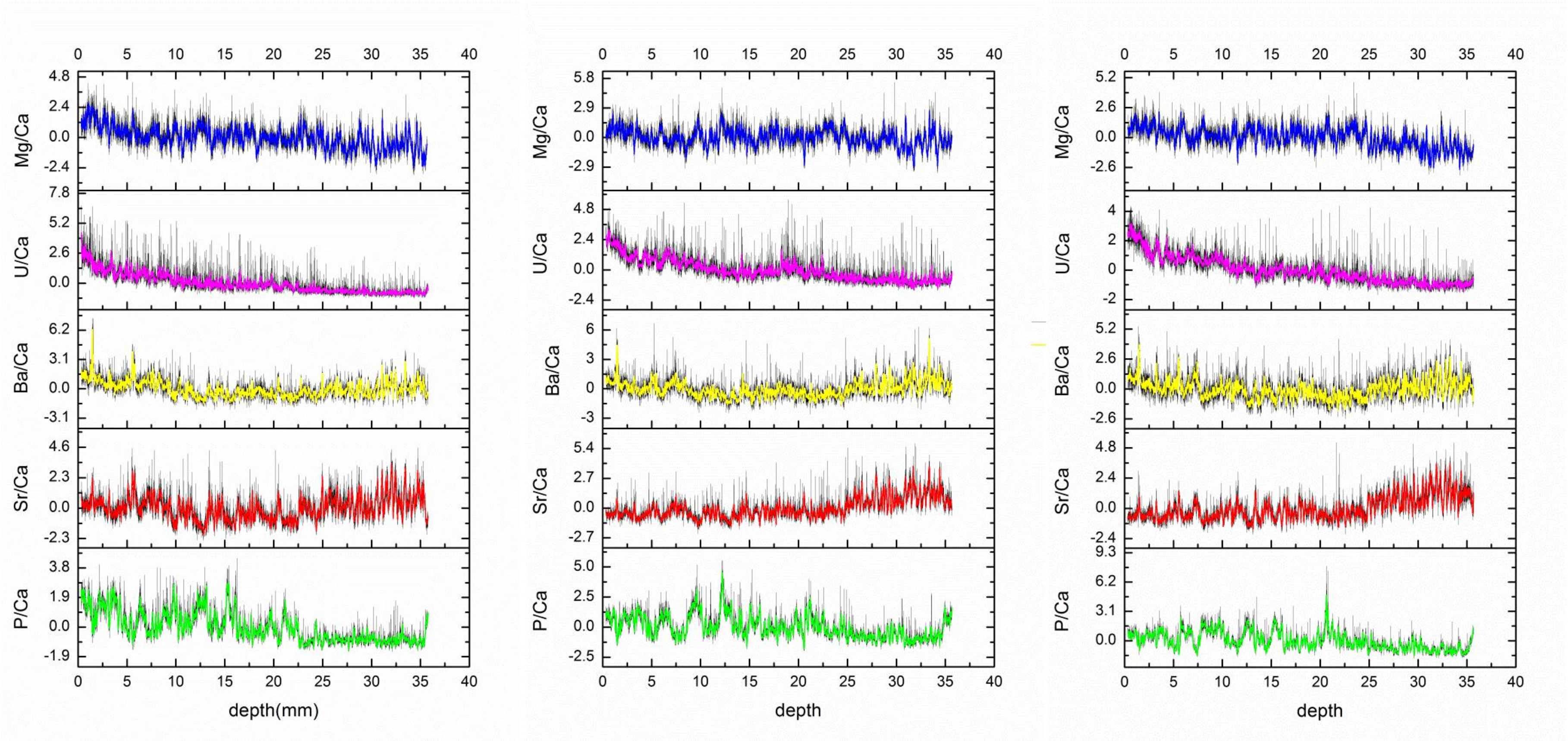

Figure S-1 The variation of P/Ca, Sr/Ca, Ba/Ca, U/Ca and Mg/Ca in the three transects. The similar annual cycles of smoothing lines are showed. (The black lines represent raw data of ion/Ca. The colourful bold lines are smoothing lines (17 point window Savitzky-Golay method).

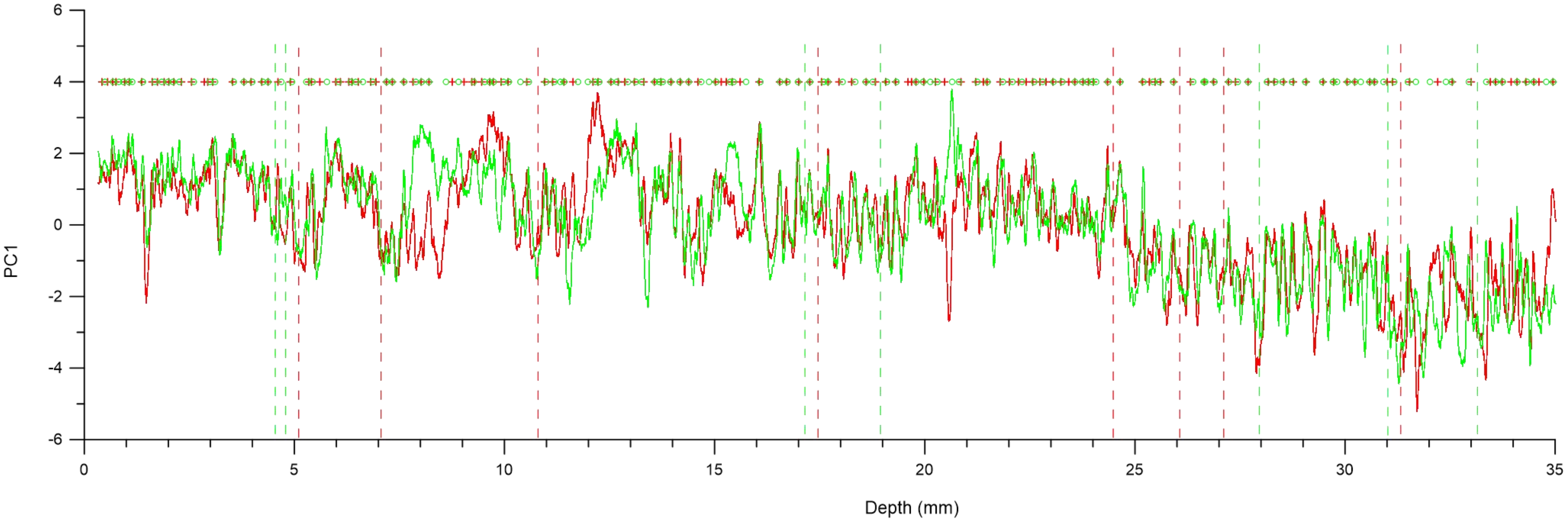

Figure S-2 A comparison between peaks identified using PCA experiments in transect 2(red line) and transect 3(green line). Red dash line (eight) and green dash line (seven) represent the removed peaks in transect 2 and 3 that only occurred in one transect with a height less 30% of the neighbouring relative peaks.
